# An Efficient Microwave Synthesis of 3-Acyl-5-bromoindole Derivatives for Controlling *Monilinia fructicola* and *Botrytis cinerea*

**DOI:** 10.3390/ijms26189148

**Published:** 2025-09-19

**Authors:** Valentina Silva, Evelyn Muñoz, Katy Díaz, Paula Molina, Ximena Besoain, Iván Montenegro, Daniela Rigano, Nelson Caro, Alejandro Madrid

**Affiliations:** 1 Laboratorio de Productos Naturales y Síntesis Orgánica (LPNSO), Facultad de Ciencias Naturales y Exactas, Universidad de Playa Ancha, Leopoldo Carvallo 270, Playa Ancha, Valparaíso 2340000, Chile; silvapedrerosv@gmail.com (V.S.); evdmunoz@gmail.com (E.M.); 2 Laboratorio de Pruebas Biológicas, Departamento de Química, Universidad Técnica Federico Santa María, Av. España N1680, Valparaíso 2340000, Chile; katy.diaz@usm.cl (K.D.); pamolinae@gmail.com (P.M.); 3 Escuela de Agronomía, Facultad de Ciencias Agronómicas y de los Alimentos, Pontificia Universidad Católicade Valparaíso, San Francisco s/n La Palma, Quillota 2260000, Chile; ximena.besoain@pucv.cl; 4 Center of Interdisciplinary Biomedical and Engineering Research for Health (MEDING), Escuela de Obstetricia y Puericultura, Facultad de Medicina, Universidad de Valparaíso, Angamos 655, Reñaca, Viña del Mar 2520000, Chile; ivan.montenegro@uv.cl; 5 Department of Pharmacy, School of Medicine and Surgery, University of Naples Federico II, Via Domenico Montesano 49, 80131 Naples, Italy; drigano@unina.it; 6 Centro de Investigación Austral Biotech, Facultad de Ciencias, Universidad Santo Tomás, Avda. Ejército 146, Santiago 8320000, Chile; ncaro@australbiotech.cl

**Keywords:** microwave-assisted synthesis, indole derivatives, filamentous fungi, molecular docking

## Abstract

Molecules containing indole cores are of interest due to their multiple biological applications and seem to be an alternative to face contemporary challenges in agriculture, especially the control of phytopathogens that affect fruit quality. In this research, starting from 5-bromoindole (**A**), eleven 3-acyl-5-bromoindole derivatives with linear (**B**–**G**) and aromatic (**H**–**L**) substitutions were obtained through microwave-assisted synthesis. The antifungal capacity of indole, 5-bromoindole (**A**) and their derivatives (**B**–**L**) were determined by in vitro assays on *Monilinia fructicola* and *Botrytis cinerea*, and a molecular docking was performed on mitochondrial complex II, succinate dehydrogenase (SDH). The results indicate that compounds **A**, **B**, **G**, and **L** were able to inhibit the growth of *M. fructicola*, while only **A** and **J** showed activity against *B. cinerea*. The acylation of **A** improved their ability to inhibit the germination of conidia on both pathogens. Compounds **A**, **B** and **G** are the most promising candidates for future research due to their inhibition on *M. fructicola* and/or *B. cinerea* and demonstrate favorable binding energies with SDH.

## 1. Introduction

Interest in molecules with indole-containing scaffolds is motivated by their remarkable bioactivity. These structures are present in plants and fungi as essential compounds like the amino acid tryptophan, the neurotransmitter serotonin, and the phytohormone indole-3-acetic acid [1]. Interestingly, the indole core nucleus can also be found in natural products of marine origin, in these, we can find a special group of halogenated indoles, such as bromoindols [2]. These molecules are versatile templates for drug development and their synthetic and natural derivatives demonstrate a range of biological activities, particularly in anticancer applications, stemming from their diverse mechanisms of action [3]; however, a variety of analgesic, antituberculosis, antiviral and antimicrobial derivatives have also been synthesized [4,5,6]. This broad pharmacological spectrum underscores the biological potential of the indole scaffold, positioning it as a promising alternative for the development of new bioactive agents to address current global challenges.

In this context, one of the most pressing challenges today is the urgent need for novel antimicrobials that are not only more effective but also less toxic, a necessity that is especially critical for controlling phytopathogens threatening food safety, crop quality, and agricultural productivity [7]. Among the most destructive diseases in agriculture are fruit rot, primarily caused by filamentous fungi such as *Monilinia fructicola* and *Botrytis cinerea*. These pathogens infect a wide range of host plants, including crops of significant commercial and nutritional value, the economic impact of these fungi is considerable, with annual losses estimated in the millions of dollars [8,9]. Moreover, controlling these pathogens has become increasingly challenging due to the emergence of resistance to conventional fungicides [10,11].

The scientific literature highlights the potential of indole derivatives as antifungal agents, particularly in the context of developing agrochemicals for the control of *Botrytis cinerea* [12]. In addition, several *N*-substituted indole derivatives have been evaluated for their antifungal activity against fungal strains of *Candida albicans*, known for their clinical significance [13]. Among the various positions in the indole structure, the substitution at C-3 position has shown promising antifungal activity, particularly against phytopathogens such as *Botrytis allii* and *Cladosporium cucumerinum*, which are responsible for significant crop damage [14]. Concerning 3-acylindole, they have been conveniently obtained through widely known synthetic methods, but new technologies open an opportunity in the design of indole derivatives, among these, microwave-assisted synthesis represents advantages such as drastic reduction in reaction time and better yields [15]. In the literature, to achieve the 3-acylation of indoles, the authors report Friedel–Crafts acylation using Lewis acids; however, due to the inconvenient presence of components with high sensitivity to moisture, it must be carried out under strictly anhydrous conditions, to overcome these difficulties, the use of alumina and modified zeolite has been explored [16,17]. Among the different strategies that have been employed to optimize this reaction, the use of a microwave-assisted catalysis system containing metal triflates dissolved in ionic liquids stands out for its regioselectivity and reusability of the system, without significant loss of catalytic activity [18].

Due to the existing background on the bioactivity of bromoindole and taking profit of the advances developed in the synthesis strategies of indole derivatives, in this study we explored the regioselective acylation of 5-bromoindole (**A**) by microwave-assisted Friedel–Crafts acylation under solvent-free conditions, to obtain a total of eleven 3-acyl-5-bromoindole derivatives (**B**–**L**) and determine their antifungal capacity against *M. fructicola* and *B. cinerea*, in addition, a molecular docking was performed to explore the potential interactions between succinate dehydrogenase and the synthesized compounds.

## 2. Results and Discussion

### 2.1. Synthesized Compounds

**Scheme 1:**
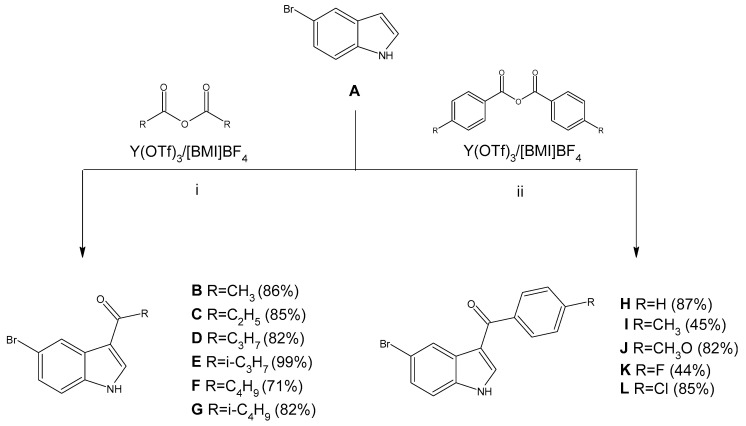
Synthesis of 5-bromoindole (**A**) derivatives with linear (**B**–**G**) and aromatic substitutions (**H**–**L**). **i**. MW, 90 °C, 150 W, 15 min. **ii**. MW, 120 °C, 150 W, 30 min.

Through microwave-assisted acylation of **A** using a series of anhydrides and the Y(OTf)_3_/[BMI]BF_4_ system as a catalyst, eleven 3-acyl-5-bromoindole derivatives (**B**–**L**) were obtained, six of which contain linear (**B**–**G**) and five are aromatic (**H**–**L**) substitutions. The synthesis methodology and yield percentages are illustrated in Scheme 1.

The yields obtained for the synthesized compounds ranged from 44% to 99%, derivatives containing linear substitutions (**B**–**G**) obtained higher yields than derivatives with aromatic substitutions (**H**–**L**). Among the linear derivatives, compound **E** was obtained in 99% yield, while among the aromatic derivatives the highest yield was obtained for compound **H**, which achieved an 87% yield. Previous research has investigated the microwave-assisted regioselective acylation of indoles, and the reported yields were lower than those achieved in our study. For example, compounds **B**, **C**, **D** and **E** were obtained with respective yields of 83%, 85%, 78% and 80% in previous studies [18], and the yields obtained in our investigation differed by a few more percentage points. One key difference between our methodology and previous studies is the microwave irradiation time, while other researchers employed shorter irradiation durations (5–10 min), and our experiments extended reaction times. Therefore, the enhanced yields observed in our study indicate that optimizing the microwave irradiation period is crucial for maximizing the efficiency of this regioselective acylation process. Compound **F**, on the other hand, had been previously reported with a yield of 20% using an acyl chloride as acylating agent [19].

The synthesis of 3-acylindoles bearing aromatic substituents has been less explored, particularly when using anhydrides as acylating agents. Generally, previous investigations have focused on obtaining these compounds using acyl chlorides. For instance, compounds **H**, **I**, **J** and **K** were previously reported to be synthesized via alumina-catalyzed acylation, achieving yields of 88%, 92%, 94% and 45%, respectively [16,20]. For this case, the yields obtained with the aforementioned methodology are higher; however, our study explores the possibility that they can also be obtained using anhydrides. On the other hand, derivative **L** had also been reported previously, obtained with a yield of 62%. [21]

Furthermore, to the best of our knowledge, the synthesized compounds **F**, **I**, **J**, **K** and **L** have not been obtained using their respective anhydrides.

### 2.2. Antifungal Activity

The effects of the compounds on the growth of *M. fructicola* and *B. cinerea* are in Table 1. In this study, the antifungal capacity of the starting compound **A**, its linear (**B**–**G**) and aromatic (**H**–**L**) derivatives, and indole were determined to evaluate the effect of bromine on antifungal activity. The results revealed that the presence of bromine at the C-5 position enhanced the indole activity against *M. fructicola* by 7.9-fold, and for *B. cinerea*, the activity of 5-bromoindole increased 13.8-fold versus indole. However, the conidia inhibition capacity remained at 5% or decreased, respectively. Given the potential of **A** over indole in mycelial inhibition, the synthesized derivatives seek to enhance its activity. The activity of the synthesized compounds is described below for each fungus.

First, for *M. fructicola*, the results showed that five compounds (indole, **A**, **B**, **G** and **L**), managed to inhibit the growth of the fungi at concentrations below 250 µg/mL, the rest of the compounds did not have this capacity. Compound **A** achieved an EC_50_ value of 7.91 ± 0.11 µg/mL, being the most effective compound among those tested, however the inhibition of conidia only reached 5%. Although the modifications made to the molecules did not improve the effectiveness of the compounds in inhibiting the growth of *M. fructicola*, they did improve the inhibition of its conidial. New perspectives in the search for agents against filamentous fungi highlight the complexity of these fungi compared to bacteria or yeasts, since they have life cycles with different morphotypes, and emphasize that to define the action of a drug specific to any species of filamentous fungus, studies should be performed on conidia [22]. Therefore, in the case of *M. fructicola*, it is important to note the improvement in the conidial inhibition of compounds **B**, **G** and **L**, these compounds achieved conidial inhibition rates of 23%, 14% and 81%, respectively.

**Figure 1:**
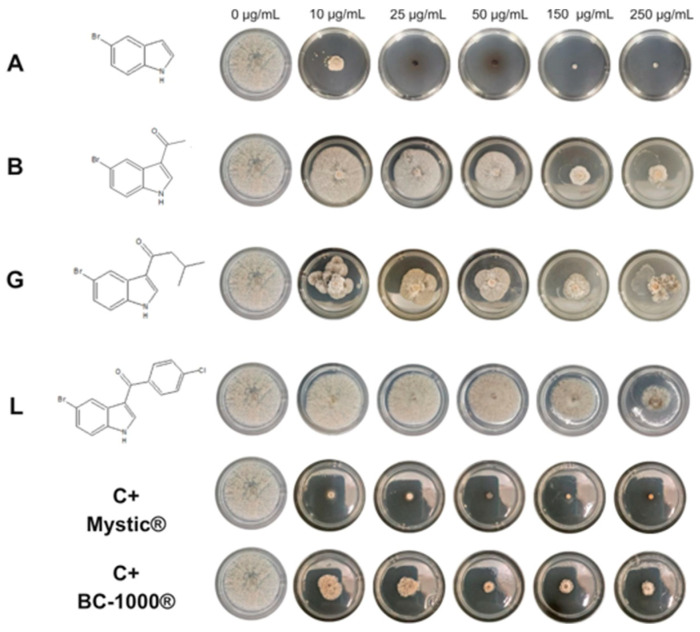
Effects of 5-bromoindole (**A**), active 3-acyl-5-bromoindole linear derivates (**B** and **G**), aromatic derivate (**L**) and positive controls (C+) on mycelial growth inhibition of *M. fructicola* in semi-solid PDA medium at different concentrations.

The 5-carbon linear acylated derivative **G** reached an EC_50_ of 36.92 ± 0.44 µg/mL in contrast to its isomer, compound **F** which was inactive. As for the aromatic acylated derivatives, only the chlorinated derivative **L** showed activity, with an EC_50_ of 240.45 ± 0.86 µg/mL, standing out with a conidial inhibition of 81%, similar to the positive controls BC-1000^®^ (natural fungicide based on grapefruit) and Mystic^®^ (antifungal formulation of compounds of the anilino-pyrimidines and oxyaminoacetates families). Effects on mycelial growth of *M. fructicola* of the active synthetized compounds are shown in Figure 1.

**Figure 2:**
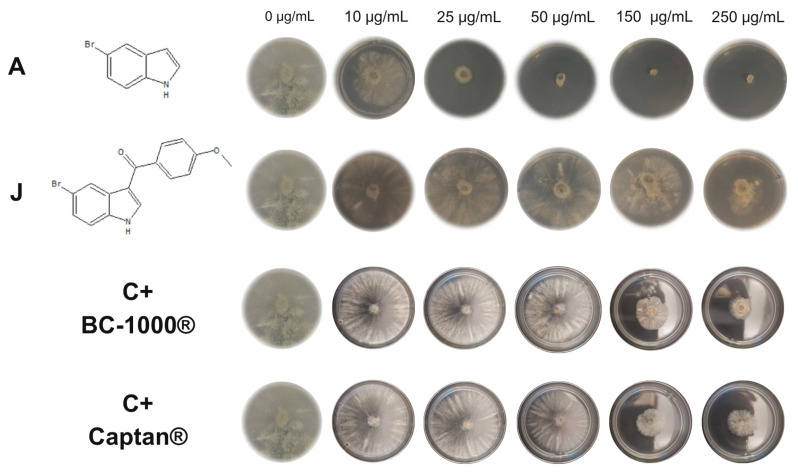
Effects of 5-bromoindole (**A**), active methoxylated aromatic derivative (**J**) and positive controls (C+) on mycelial growth inhibition of *B. cinerea* in semi-solid PDA medium at different concentrations.

For *B. cinerea*, a phenomenon similar to that observed for *M. fructicola* occurs. The results of the inhibition of this fungus can be found in Table 1. For this case, the compound that achieved mycelial inhibition at lower concentrations was also compound **A**, none of the other linear acylated derivatives were able to inhibit *B. cinerea* at the concentrations tested, only the methoxylated aromatic derivative **J**, reached an EC_50_ of 208.7 ± 0.83 µg/mL, the scenario is similar, since this derivative also improved its ability to inhibit the germination of conidia, reaching 63%. The literature describes that *B. cinerea* is polyphagous and has a great capacity to generate resistance, the control of this pathogen is complex due to its multiple mechanisms of pathogenicity, so management tools are constantly sought [23]. This is also evidenced in our research, note that *B. cinerea* was less susceptible to the positive controls used, including BC-1000^®^ and Captan^®^, a commercial botrycide belonging to the phthalimide chemical group. These controls achieved an EC_50_ value of 95.13 ± 0.78 µg/mL and 82.89 ± 0.69 µg/mL, respectively; therefore, compound **A** outperformed the efficacy of both positive controls., which is remarkable, reaching an EC_50_ as low as 12.17 ± 0.07 µg/mL. The effects of active compounds and positive controls are shown in Figure 2.

Other studies have explored the activity of halogenated indoles on the growth of *B. cinerea* and determined that the presence of electron-withdrawing group at position C-4 and C-5 was key to their bioactivity [24]. Similarly, our study has confirmed the great antifungal potential of 5-bromoindole over indole and shows that the modifications made could improve the ability to inhibit conidia germination. They are also valuable thanks to the reactivity of the acyl group, which allows for a wide variety of chemical transformations in which they can be a starting point for new syntheses or key intermediates.

Regarding the decrease in mycelial inhibition in some derivatives, it is suggested that these substitutions may interfere with the binding site or alter their ability to form bonds with biological targets of filamentous fungi. This effect has also been described by Dhimanyin et al. in the case of substitutions with aldehydes or olefins at the C-3 position of different indoles, where these substitutions caused a significant reduction in anticancer activity [25].

As a result of in vitro testing, it is concluded that compound **A** has a high capacity to inhibit the mycelium of both pathogens and, together with compounds **B**, **G**, **J**, and **L**, are positioned as an alternative for the development of controls for these phytopathogens, due to its capacity to inhibit mycelium and conidia germination. Just as there is commercial antifungal formulations composed of more than one active ingredient, such as Mystic^®^, which contains pyrimethanil and trifloxystrobin, this strategy could be applied to improve effectiveness, generating formulations containing compound **A** and compound **L**, thanks to their great capacity to inhibit the mycelium and conidia of *M. fructicola*, respectively. In future research, the potential of formulations containing compounds **A** and **J** could be explored, as the same phenomenon occurs on *B. cinerea*.

### 2.3. Molecular Docking

Succinate dehydrogenase (SDH), also referred to as mitochondrial complex II, is an enzyme embedded in the inner mitochondrial membrane, playing a central role in both the tricarboxylic acid (Krebs) cycle and the electron transport chain. Due to its critical function in cellular respiration, SDH has become a strategic target for the development of fungicides. Its inhibition disrupts fungal energy metabolism by blocking the transfer of electrons from succinate to ubiquinone, effectively halting the respiratory chain [26,27,28,29].

In this study, molecular docking analyses were carried out to explore the potential interactions between SDH and a series of indole-based derivatives. Preliminary docking calculations were also carried out on sterol 14-α-demethylase (CYP51) from *Candida albicans* (PDB ID: 5TZ1, 2.0 Å resolution). In contrast to the strong affinities observed with SDH, the binding energies against CYP51 were markedly lower (ranging from −2.18 to −4.78 kcal/mol). These results showed poor correlation with the experimental antifungal activity (Table 2), further supporting SDH as the most suitable molecular target for our compounds.

The docking simulations were based on the crystallographic structure of SDH available under PDB ID: 2FBW. The compound 2-methyl-N-phenyl-5,6-dihydro-1,4-oxathiine-3-carboxamide, the ligand co-crystallized in 2FBW, was employed as a reference molecule for comparative analysis. Molecular docking simulations were performed to evaluate the binding affinity and interaction profiles of a series of indole-based compounds with succinate dehydrogenase (SDH), using the crystallographic structure (PDB ID: 2FBW) as the receptor model. The co-crystallized ligand 2-methyl-N-phenyl-5,6-dihydro-1,4-oxathiine-3-carboxamide (CBE) served as a reference and yielded a docking score of −7.2 kcal/mol, consistent with its known high binding affinity.

Among the tested compounds, compound **A** exhibited the most favorable binding energy (−6.81 kcal/mol), closely approaching that of the reference ligand. This compound formed key hydrogen bonds and hydrophobic interactions with residues Asp232, Ser229, Lys49, and Val24, located within the active site pocket. Compound **G** also showed strong affinity (−6.79 kcal/mol) and engaged several residues including Asp232, Thr48, Ala178, Phe177, Lys49, and Val24.

Compound **B** displayed moderately high binding energies of −6.56 kcal/mol, interacting primarily with conserved residues such as Leu50, Lys49, and Gly27, which are involved in the stabilization of ligand binding. In contrast, compounds **C**, **D**, **E**, **F**, **H**, **I**, **J**, **K** and **L** exhibited lower binding affinities, ranging between −5.07 and −5.98 kcal/mol, although most still retained interactions with the catalytically relevant residue Asp232.

**Figure 3:**
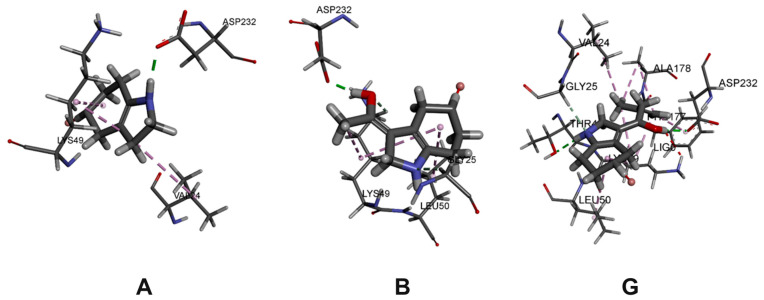
Three-dimensional predicted binding mode of compounds **A**, **B**, and **G** into the allosteric site of enzyme SDH (PDB: 2FBW).

The activity of the compounds on SDH is related to the type of molecular interactions formed between the ligand and the amino acid residues of the protein (Figure 3). One of the most important interactions corresponds to hydrogen bonds; a higher number of hydrogen bonds of this type would apparently be related to higher activity [30]. Compound **A**, the most active in the series, established a conventional hydrogen bond with residue Asp232 at 1.98 Å. Similarly, compounds **B** and **G** also formed hydrogen bonds with the same residue, at distances of 2.01 Å and 2.05 Å, respectively. Although the remaining compounds also exhibited this type of interaction, the analyses revealed the presence of repulsive interactions, which could compromise the stability of the formed complexes and account for their lower docking energies [31].

It should also be noted that the structure–activity relationship in this compound series was not straightforward. While most derivatives established contacts with Asp232, variations in binding geometry and the presence of destabilizing interactions likely account for the irregular activity trends, a behavior that has also been described for other SDH fungicides [32].

Several studies have highlighted the potential of indole-based derivatives as inhibitors of SDH, although the specific compounds evaluated in this study have not been previously described as SDH inhibitors, structurally related molecules have shown promising biological activity. Indole- and benzo-fused heterocyclic carboxamides have been shown to effectively inhibit SDH activity in vitro, suggesting that the indole scaffold may serve as a valuable pharmacophore for the development of new SDH-targeting agents [33]. These findings support the rationale for further investigating these compounds in the context of SDH inhibition and antifungal activity.

Overall, the docking results indicate that compounds **A**, **B** and **G** are the most promising candidates for further investigation, as they demonstrate favorable binding energies and interaction patterns like the co-crystallized inhibitor CBE.

## 3. Materials and Methods

### 3.1. General

All chemicals and reagents were obtained from Sigma-Aldrich Co. (St. Louis, MO, USA) and AK Scientific Inc. (Union City, CA, USA) and were used without further purification. Reactions were monitored by thin layer chromatography (TLC) on TLC precoated silica gel 60 F254 plates (Merck KGaA, Darmstadt, Germany). NMR spectra were measured on a Bruker Avance 400 Digital NMR spectrometer (Bruker Corporation, Billerica, MA, USA) in acetone with TMS as the internal standard. Chemical shifts (δ) are reported in ppm and coupling constants (*J*) are expressed in Hertz.

### 3.2. Synthesis of Compounds ***B**–**L***

Microwave assisted synthesis of derivatives **B**–**L** were performed in a CEM Discover 2.0 microwave synthesizer (Matthews, NC, USA), using high-temperature, high-pressure, sealed reaction vessels of 10 mL. Vessels were charged with 5-bromoindole, [BMI]BF_4_, Y(OTf)_3_ and the corresponding anhydride in a 1:1:0.01:1 mole ratio. Reactions were performed using standard mode, constant stirring at high intensity and the power output was set to 150 W. Temperature and reaction time were adjusted as suggested by Tran et al. with slight modifications [18]. Then, the vessel was cooled down to 50 °C by rapid compressed air cooling and the mixture was extracted with ethyl acetate and washed in with a solution of NaHCO_3_ (3 × 30 mL) and brine (3 × 15 mL). The obtained yield was determined after precipitation of the compounds with diethyl ether or purification by column chromatography.

All the synthesized compounds were analyzed spectroscopically. Their data were contrasted with the information available in the literature, and their respective spectra are included in the Supplementary Material. The details for all compounds are described below; however, compounds **F**, **K**, and **L**, which were synthesized for the first time using this protocol, are described in more detail in this section.

1-(5-bromo-1H-indol-3-yl)ethan-1-one (**B**): The compound was isolated as a light brown solid with a yield of 86%. f.p.: 224 °C. NMR data of **B** was consistent with that reported in the literature [18].

1-(5-bromo-1H-indol-3-yl)propan-1-one (**C**): The compound was isolated as a white solid with a yield of 85%. f.p.: 232 °C. NMR data of **C** was consistent with that reported in the literature [18].

1-(5-bromo-1H-indol-3-yl)butan-1-one (**D**): The compound was isolated as a white solid with a yield of 82%. f.p.: 213 °C. NMR data of **D** was consistent with that reported in the literature [18].

1-(5-bromo-1H-indol-3-yl)-2-methylpropan-1-one (**E**): The compound was isolated as a white solid with a yield of 99%. f.p.: 199–200 °C. NMR data of **E** was consistent with that reported in the literature [18].

1-(5-bromo-1H-indol-3-yl)pentan-1-one (**F**): The compound was isolated as a light pink solid with a yield of 71%. f.p.: 165–166 °C. ^1^H NMR (400 MHz, CD_3_COCD_3_): δ 8.52 (s, 1H, H-4); 8.28 (s, 1H, H-2); 7.46 (d, *J* = 8.6 Hz, 1H, H-7); 7.34 (dd, *J* = 2.0 and 8.6 Hz, 1H, H-6); 2.86 (t, *J* = 7.4 Hz, 1H, H-2′); 1.69 (m, 2H, H-3′); 1.39 (m, 2H, H-4′); 0.92 (t, *J* = 7.4 Hz, 1H, H-5′). ^13^C NMR (100 MHz, CD_3_COCD_3_): δ 196.4 (C-1′); 137.2 (C-8); 131.9 (C-2); 127.2 (C-9); 126.5 (C-6); 123.3 (C-4); 117.6 (C-3); 116.1 (C-5); 112.9 (C-7); 39.6 (C-2′); 26.8 (C-3′); 22.2 (C-4′); 13.3 (C-5′).

1-(5-bromo-1H-indol-3-yl)-3-methylbutan-1-one (**G**): The compound was isolated as a white solid with a yield of 82%. f.p.: 211 °C. NMR data of **G** was consistent with that reported in the literature [18].

(5-bromo-1H-indol-3-yl)(phenyl)methanone (**H**): The compound was isolated as a white solid with a yield of 87%. f.p.: 261–262 °C. NMR data of **H** was consistent with that reported in the literature [16].

(5-bromo-1H-indol-3-yl)(4-methylphenyl)methanone (**I**): The compound was isolated as a white solid with a yield of 45%. f.p.: 255–256 °C. NMR data of **I** was consistent with that reported in the literature [16].

(5-bromo-1H-indol-3-yl)(4-methoxyphenyl)methanone (**J**): The compound was isolated as a light brown solid with a yield of 82%. f.p.: 245–246 °C. NMR data of **J** was consistent with that reported in the literature [16].

(5-bromo-1H-indol-3-yl)(4-fluorophenyl)methanone (**K**): The compound was isolated as a white solid with a yield of 44%. f.p.: 268–269 °C. ^1^H NMR (400 MHz, CD_3_COCD_3_): δ 8.55 (s, 1H, H-4); 8.54 (s, 1H, H-2); 7.94 (m, 2H, H-3′ and H-7′); 7.53 (d, *J* = 8.6 Hz, 1H, H-7); 7.41 (dd, *J* = 2.0 and 8.6 Hz, 1H, H-6); 7.30 (m, 2H, H-4′ and H-6′). ^13^C NMR (100 MHz, CD_3_COCD_3_): δ 189.0 (C-1′); 165.1(C-5′); 137.6 (C-8); 136.3 (C-2′); 131.7 (C-3′ and C-7′); 129.1 (C-9); 126.7 (C-6); 124.9 (C-2 and C-4); 115.7 (C-4′ and C-6′); 114.4 (C-7).

(5-bromo-1H-indol-3-yl)(4-chlorophenyl)methanone (**L**): The compound was isolated as a light yellow solid with a yield of 85%. f.p.: 252–253 °C. ^1^H NMR (400 MHz, CD_3_COCD_3_): δ 8.55 (s, 1H, H-4); 8.04 (s, 1H, H-2); 7.86 (m, 2H, H-3′ and H-7′); 7.56 (m, 3H, H-4′, H-6′ and H-7); 7.41 (dd, *J* = 2.0 and 8.6 Hz, 1H, H-6). ^13^C NMR (100 MHz, CD_3_COCD_3_): δ 189.1 (C-1′); 139.8 (C-5′); 137.2 (C-2′); 136.6 (C-8); 130.8 (C-4′ and C-6′); 129.1 (C-3′ and C-7′); 126.7 (C-6 and C-9); 124.9 (C-2 and C-4); 115.8 (C-3 and C-5); 114.5 (C-7).

### 3.3. In Vitro Antifungal Activity of Indol and Synthetized Compounds (***A**–**L***) Against Phytopathogenic Fungi

#### 3.3.1. Fungal Growing Conditions

The isolates of *M. fructicola* y *B. cinerea* used were obtained from affected nectarines and vid from commercial orchards in the Metropolitan Region, Chile. These isolates were transferred and identified thanks to the collaboration of the mycology unit and molecular biology laboratories of the Servicio Agrícola y Ganadero (SAG), Chile. Potato dextrose agar (PDA; DIFCO) was used as culture medium, and the fungi were incubated for 9 days at 23 °C. Conidia Suspensions (1 × 10^5^ spores/mL) were incubated in a potato dextrose agar medium between 5 and 7 days at 23 °C for both pathogens.

#### 3.3.2. Effect of the Compounds on the Micelial Growth of *M. fructicola* and *B. cinerea* In Vitro

Antifungal activity of indol, 5-bromoindol (**A**), their synthetic derivatives (**B**–**L**), and of commercial fungicide BC-1000^®^ (CHEMIE Research & Manufacturing, Casselberry, FL, USA), Mystic^®^ 520 SC (Lot: PAIS004727; Bayer, Santiago, Chile) and Captan^®^ 13W (ANASAC CHILE S.A., Santiago, Chile), were assessed using the radial growth test at final concentrations of 0, 10, 25, 50, 150 and 250 µg/mL in PDA medium [34]. The amount of added ethanol (1%) was identical in negative control and treatment assays. For each treatment, a 4 mm disc of mycelium was inoculated in the center of a plate and incubated for 3 (for *B. cinerea*) o 5 days (*M. fructicola*), at 23 °C in complete darkness. Treatments were performed in triplicate. Mycelial growth diameters were measured, and inhibition percentages were calculated using the standard method. The results are expressed as the average effective concentration (EC_50_), that is, the concentration at which mycelial growth was reduced by 50%. This value was determined by regression of the values of the percentage inhibition of the radial growth versus the concentration values of the compound using Origin ProV.8 software (OriginLab Corporation, Northampton, MA, USA) [35].

#### 3.3.3. Effect of the Compounds on Conidial Germination

The inhibition of conidial germination by the compounds and controls was determined following the methodology described by Ferreira et al., 2025 with modifications [34]. Spore suspensions were prepared from 9-day-old *M. fructicola* colonies grown on PDA at 23 °C, unlike *B. cinerea* that were incubated for 6 days to obtain conidia. A 40 µL aliquot of a spore suspension at a concentration of 1 × 10^5^ conidia/mL was spread with drigalsky loop on the plates with culture medium containing the compounds at different concentrations (10, 25, 50, 150 and 250 µg/mL), then incubated at 23 °C for 16 h in the dark. Sterile distilled water was used as a negative control. Conidia were considered germinated when they developed germ tubes at least twice their width. The percentage of inhibition of conidial germination (ICG%) was calculated using the following Equation (1):

ICG% = [(**CC** − **CT**)/**CC**] × 100 (1)

where **CC** is total conidia germinated in the control and **CT** correspond to total conidia germinated in the treatment. The percentage of germinated conidia by randomly examining 100 conidia in the center of each plate by microscopy (40× magnification; Leica DM500, Leica Microsystems, Wetzlar, Germany). All the measurements were carried out in triplicate.

### 3.4. Molecular Docking

Three-dimensional structures of the ligands were generated using UCSF CHIMERA version 1.18. Polar hydrogen atoms were incorporated, and Gasteiger partial charges were assigned employing the Amber ff14SB force field. Subsequently, each structure underwent energy minimization within the same platform and was exported in MOL2 format for docking analyses. Molecular graphics were rendered with the free version of Discovery Studio Visualizer (v17.2.0.16349, 2016).

The crystal structure of succinate dehydrogenase (SDH) (PDB ID: 2FBW, resolution 2.06 Å) was retrieved from the Protein Data Bank (http://www.rcsb.org/, accessed on 26 May 2025). Molecular docking simulations between SDH and the selected ligands were performed using the AutoDock4 suite, applying the Lamarckian genetic algorithm. In the simulations, the protein was treated as rigid, while ligand flexibility was fully considered.

Docking parameters included 50 independent runs, each with a maximum of 25,000,000 energy evaluations per ligand. Clustering of docking poses was performed using an RMSD cutoff of 0.5 Å. The co-crystallized ligand, 2-methyl-N-phenyl-5,6-dihydro-1,4-oxathiine-3-carboxamide (CBE), was used as a reference. The docking grid was centered at coordinates X = 20.46 Å, Y = 60.98 Å, and Z = 20.31 Å, with a grid box size of 40 points in each dimension (X, Y, and Z).

Docking outcomes were assessed based on binding free energy (ΔG) and the population of clusters, with the lowest-energy, most populated cluster selected for further analysis. Re-docking of the co-crystallized ligand under identical conditions yielded an RMSD of 2.49 Å, validating the docking protocol.

## 4. Conclusions

This study successfully synthesized eleven 3-acyl-5-bromoindole derivatives via microwave-assisted Friedel–Crafts acylation under solvent-free conditions, including the novel synthesis of compounds **F**, **K**, and **L**. Compound **A** exhibited remarkable antifungal activity against *M. fructicola* and *B. cinerea*, even surpassing commercial fungicides in controlling *B. cinerea*. While C-3 modifications seemed to reduce mycelial growth inhibition, derivatives **B**, **G**, **J**, and **L** significantly improved conidial germination inhibition. Molecular docking simulations supported the antifungal activity of compounds **A**, **B**, and **G**. The findings suggest that compounds **A**, **B**, **G**, **J**, and **L** hold promise for developing novel, effective agrochemicals to combat plant pathogens.

## Figures and Tables

**Table 1:** Mean effective concentration (EC_50_) value and inhibition of conidial germination of indole, 5-bromoindole (**A**) and the synthesized compounds **B**–**L** against *Monilinia fructicola* and *Botrytis cinerea* in vitro.

Compound	*Monilinia fructicola* (µg/mL) ± SD	Conidia ^a^ (%)	*Botrytis cinerea* (µg/mL) ± SD	Conidia ^b^ (%)
**Indole**	62.85 ± 0.08	5	168.03 ± 0.03	36
**A**	7.91 ± 0.11	5	12.17 ± 0.07	18
**B**	53.6 ± 0.54	23	>250	I
**C**	>250	I	>250	I
**D**	>250	I	>250	I
**E**	>250	I	>250	I
**F**	>250	I	>250	I
**G**	36.92 ± 0.44	14	>250	I
**H**	>250	I	>250	I
**I**	>250	I	>250	I
**J**	>250	I	208.7 ± 0.83	63
**K**	>250	I	>250	I
**L**	240.45 ± 0.86	81	>250	I
BC-1000^®^	<10	84	95.13 ± 0.67	100
Captan^®^	-	-	82.89 ± 0.69	100
Mystic^®^	<10	85	-	-

^a,b^ Inhibition percentage of conidial germination of *M. fructicola* and *B. cinerea*, respectively, obtained at the calculated EC_50_. BC-1000^®^; Captan^®^ and Mystic^®^, commercial products used as positive control. (±SD): Standard deviation value; I: no inhibitory effect. These values were estimated by measuring mycelium growth of the pathogens after 120 h y 72 h of incubation, respectively.

**Table 2:** Binding energies obtained from molecular docking studies on SDH.

Compound	Binding Energy (Kcal/mol)	Interactions
**A**	−6.81	Asp232; Ser229; Lys49; Val 24
**B**	−6.56	Asp 232; Leu50; Lys49; Gly25
**C**	−5.16	Asp232; Lys49; Leu50
**D**	−5.07	Asp232; Thr48; Ala178; Leu50; Gly25; Lys49
**E**	−5.71	Gly27; Hse56; Leu50; Gly27; Leu417
**F**	−5.08	Asp232; Thr48; Val24; Gly25; Leu50; Lys49
**G**	−6.79	Asp232; Thr48; Ala178; Phe177; Lys49; Val24
**H**	−5.68	Thr48; Thr213; Ala178; Lys49
**I**	−5.52	Ser229; Tyr365; Leu417; Asp232
**J**	−5.98	Ser413; Gly397; Glu397; Leu417
**K**	−5.43	Ser413; Gly27; Ala212; Leu 413
**L**	−5.98	Ala212; Gly27; Leu417; Leu414

## Data Availability

All data are available in the manuscript or Supplementary Material.

## Supplementary Materials

The following supporting information can be downloaded at: https://www.mdpi.com/article/10.3390/ijms26189148/s1.
