# Supplementary material for: An Efficient Microwave Synthesis of 3-Acyl-5-bromoindole Derivatives for Controlling Monilinia fructicola and Botrytis cinerea
Source: Int J Mol Sci. 2025 Sep 19;26(18):9148. doi: 10.3390/ijms26189148 (PMC12471050; doi:10.3390/ijms26189148)

SUPPORTING INFORMATION

An Efficient Microwave Synthesis of 3-acyl-5-bromoindole Derivatives for Controlling *Monilinia fructicola* and *Botrytis cinerea*.

Valentina Silva [1], Katy Diaz [2], Paula Molina [2], Evelyn Muñoz [1], Ximena Besoain [3], Iván Montenegro [4], Daniela Rigano [5], Nelson Caro [6] and Alejandro Madrid [1,*]

^{1}H NMR spectrum of compound **B**

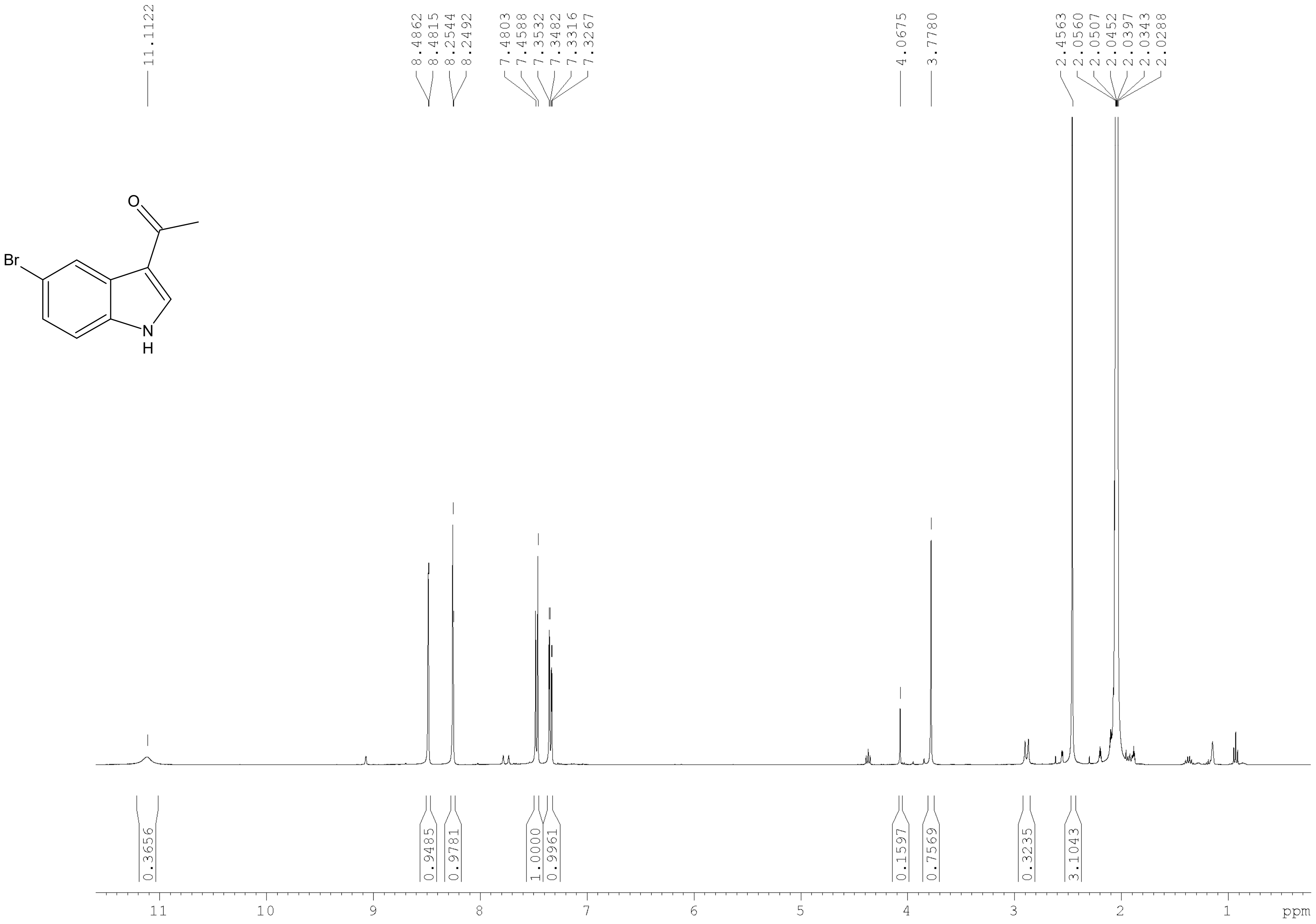

Mass spectra of compound **B**

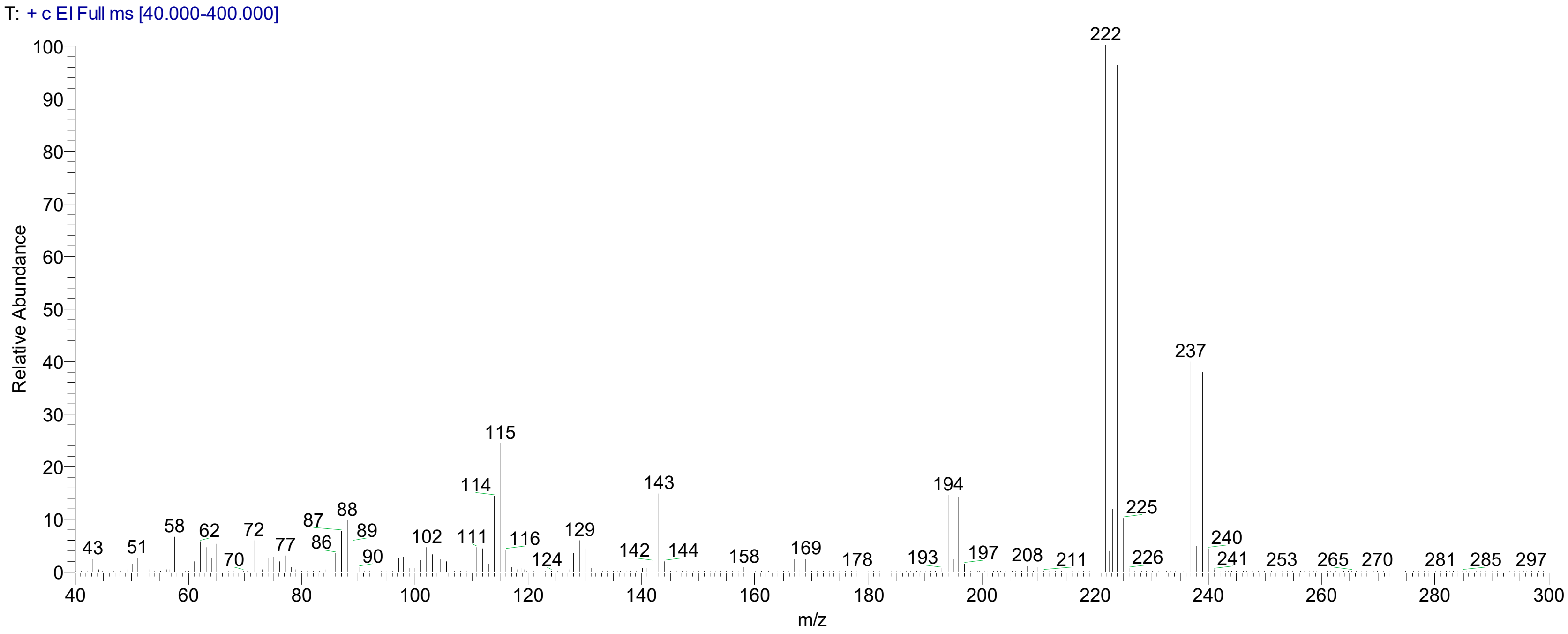

^{1}H NMR spectrum of compound **C**

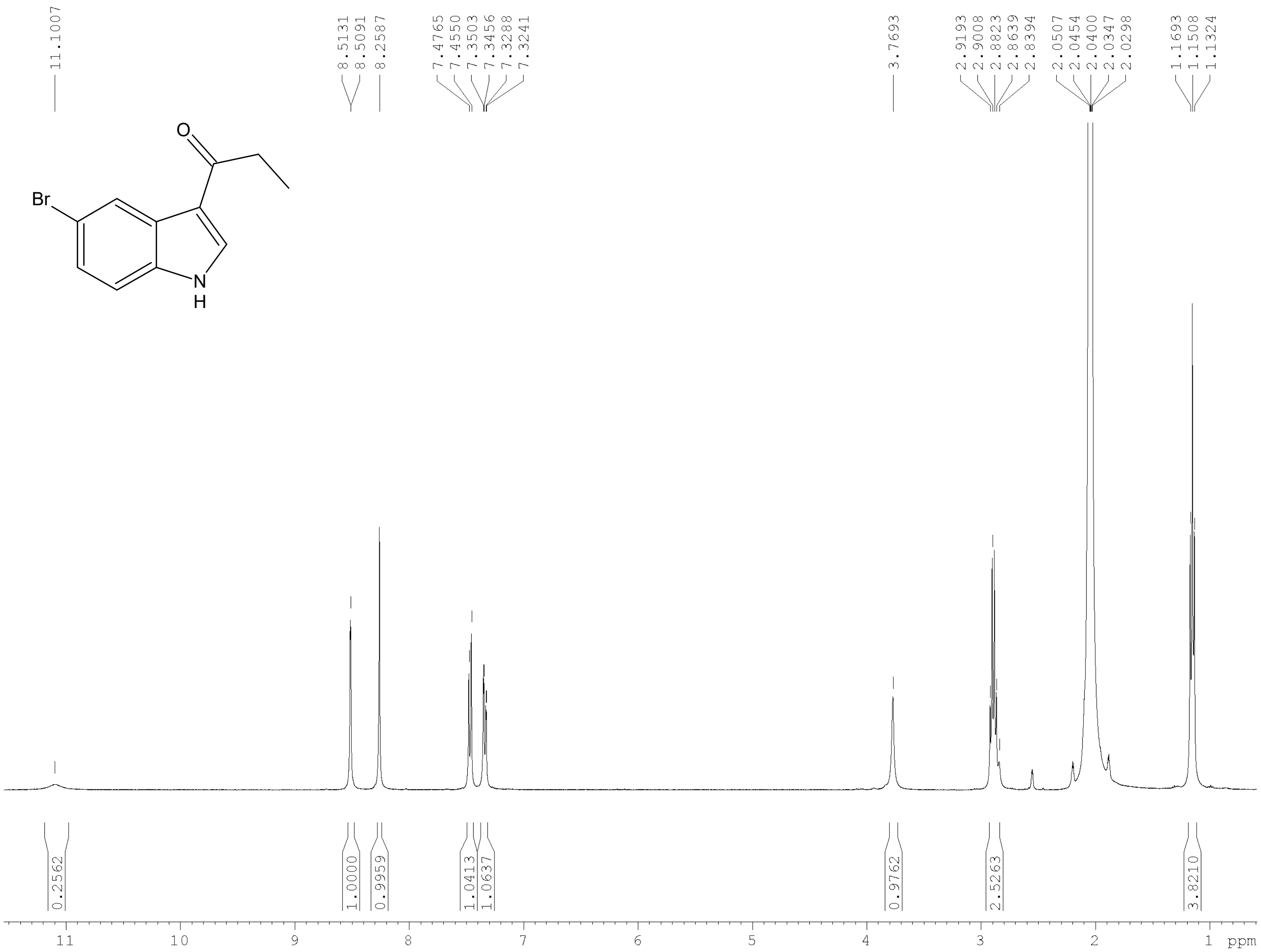

Mass spectra of compound **C**.

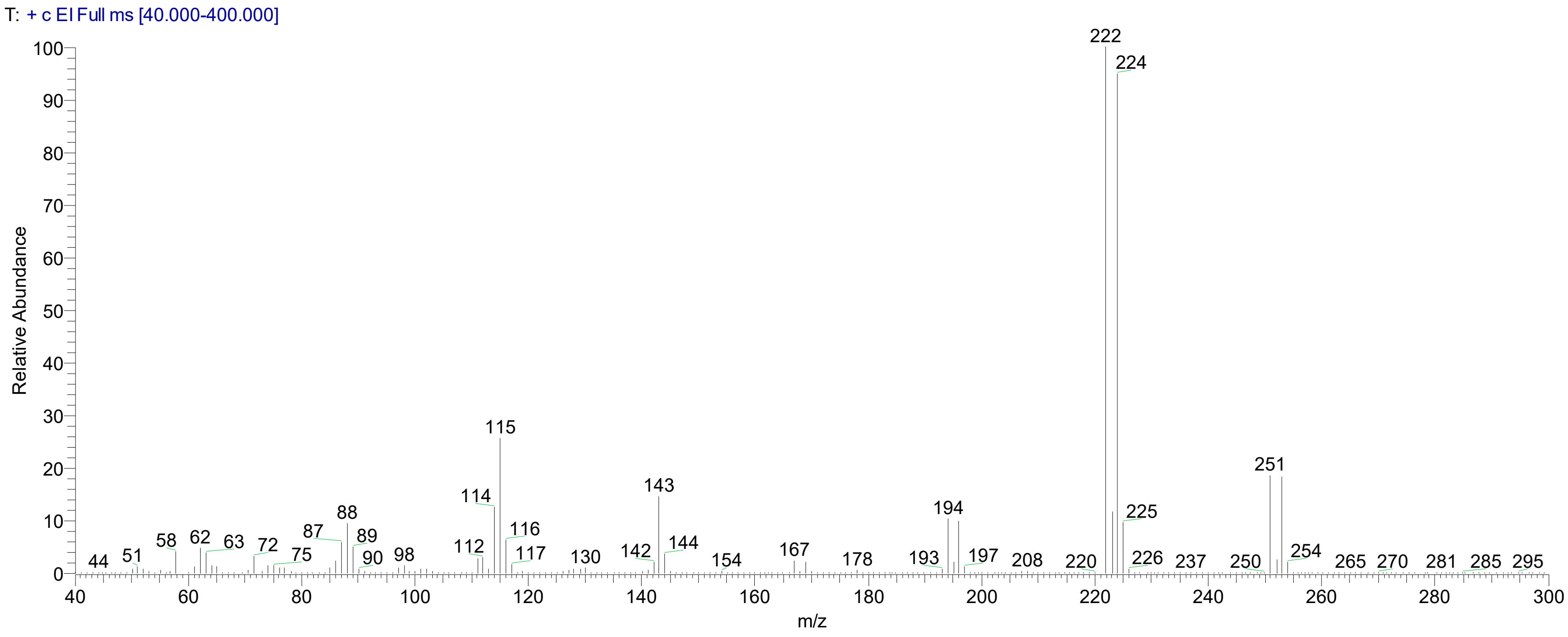

^{1}H NMR spectrum of compound **D**

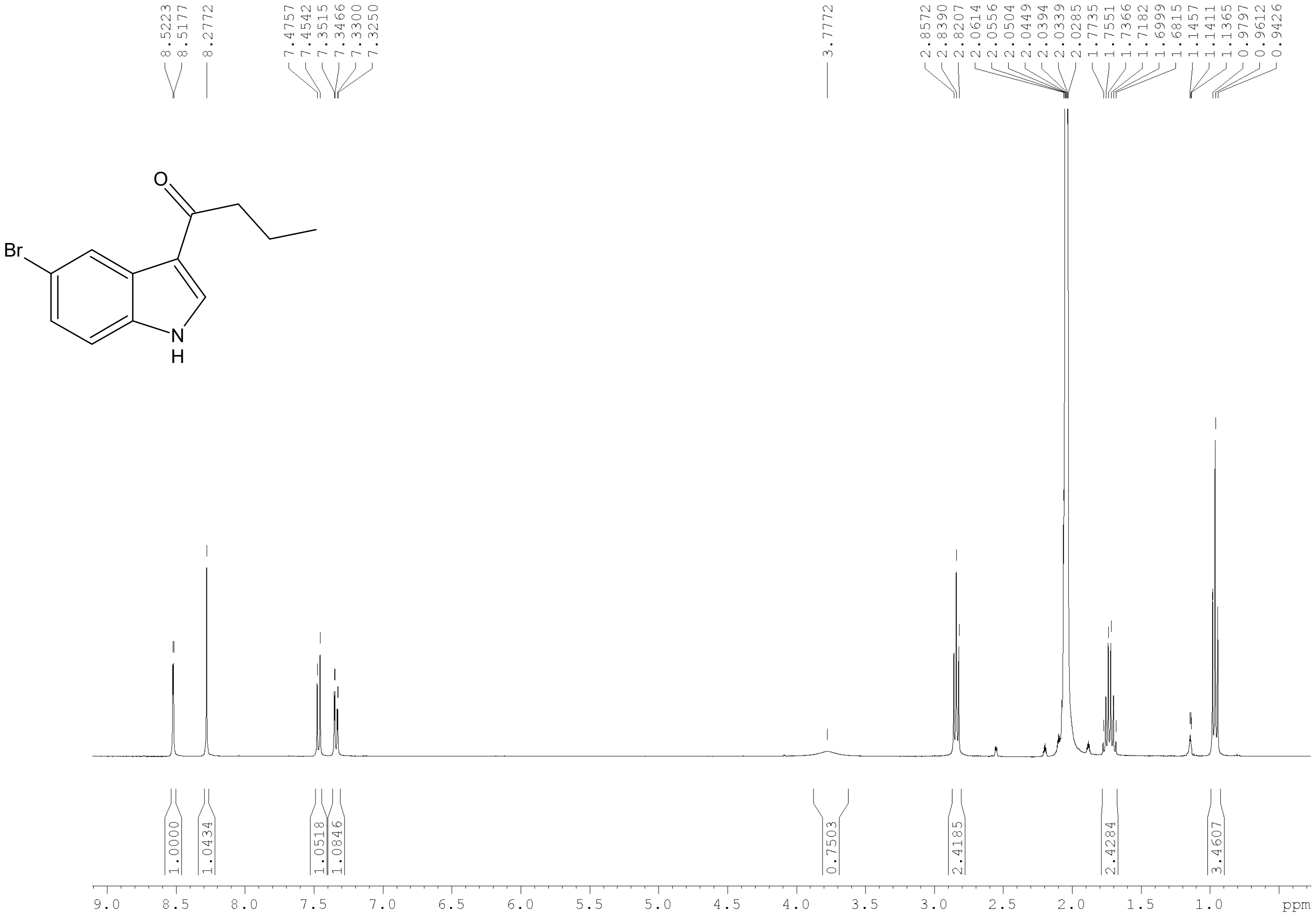

Mass spectra of compound **D**.

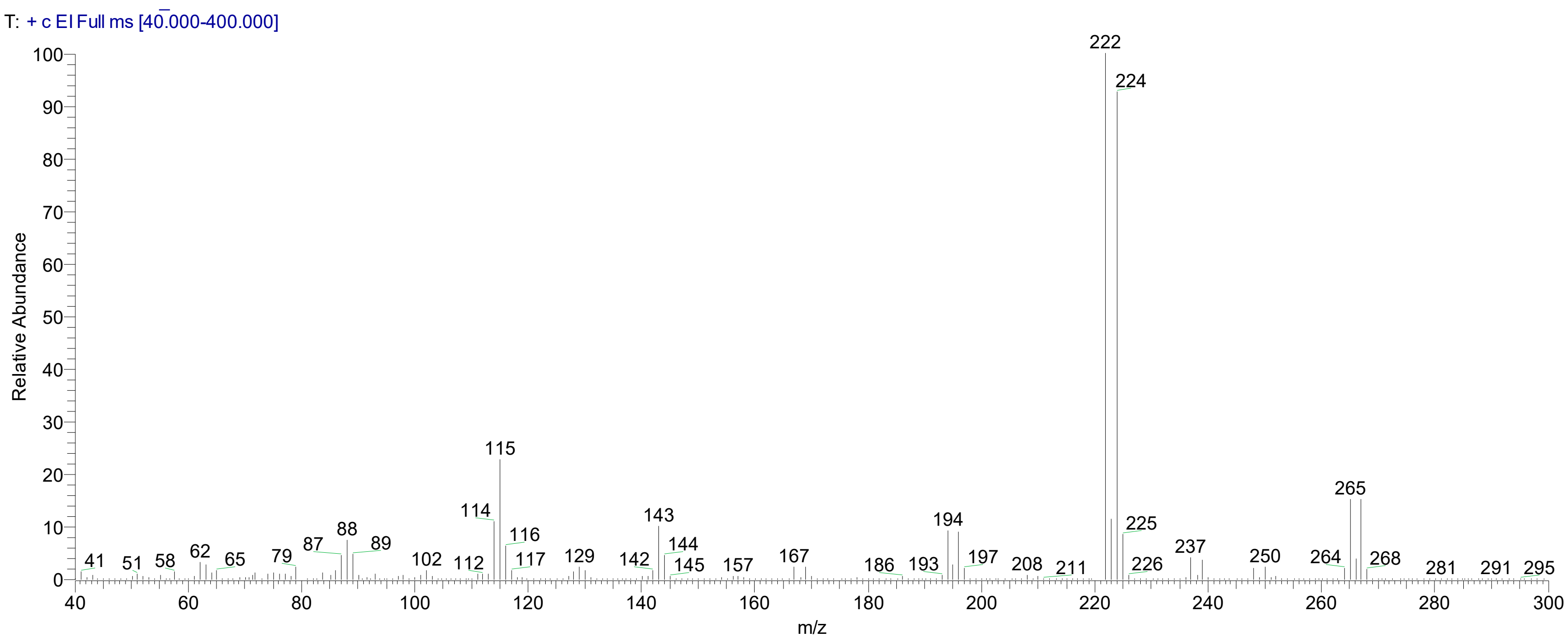

^{1}H NMR spectrum of compound **E**

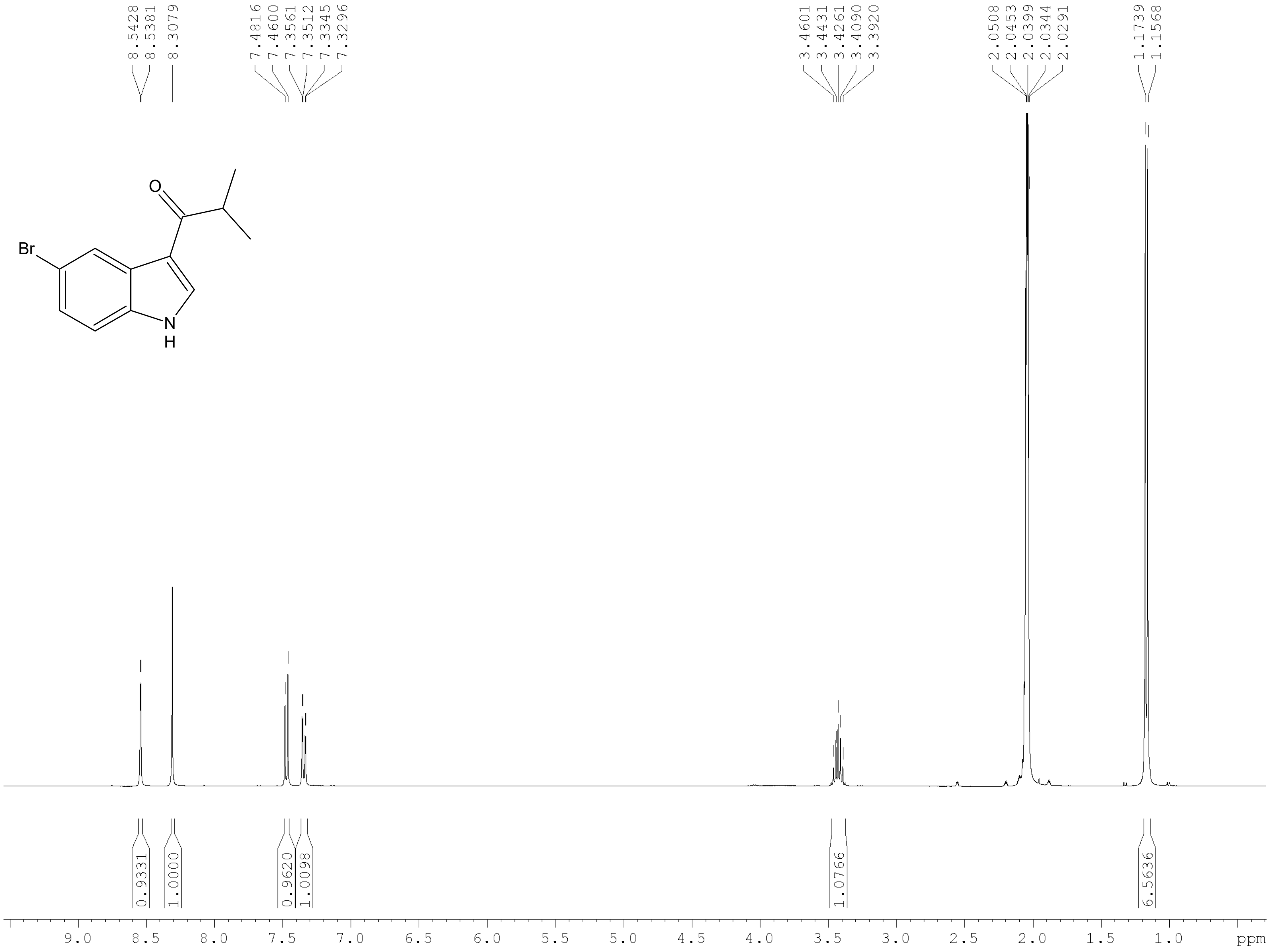

Mass spectra of compound **E**.

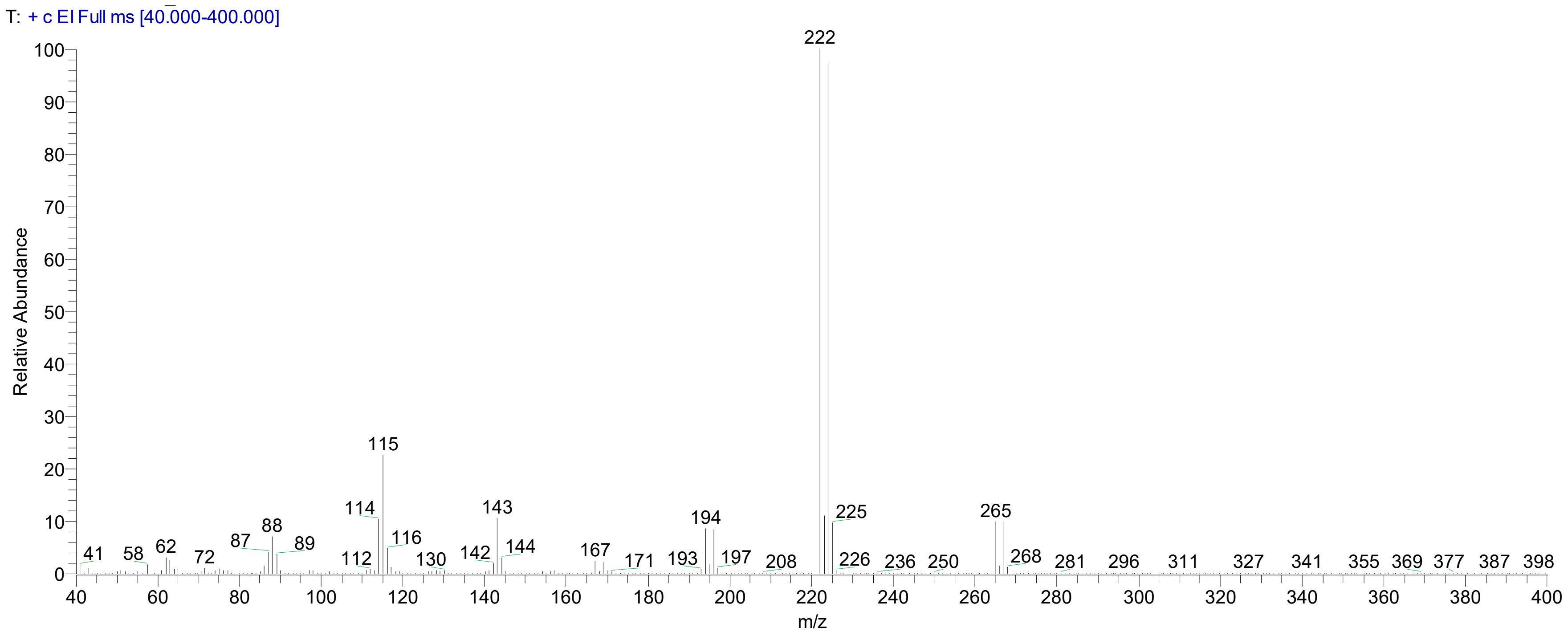

^{1}H NMR spectrum of compound **F**.

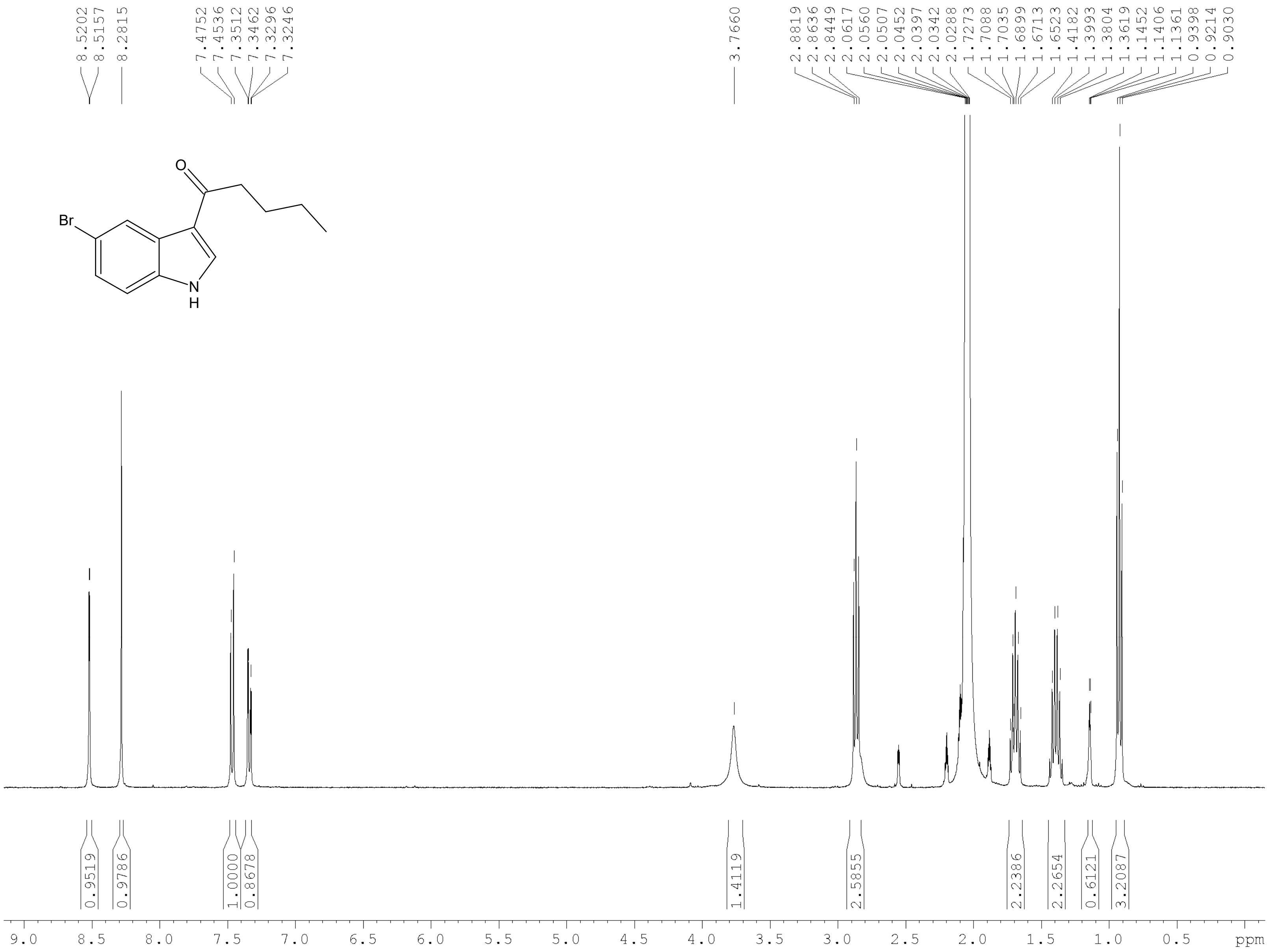

^{13}C NMR spectrum of compound **F**.

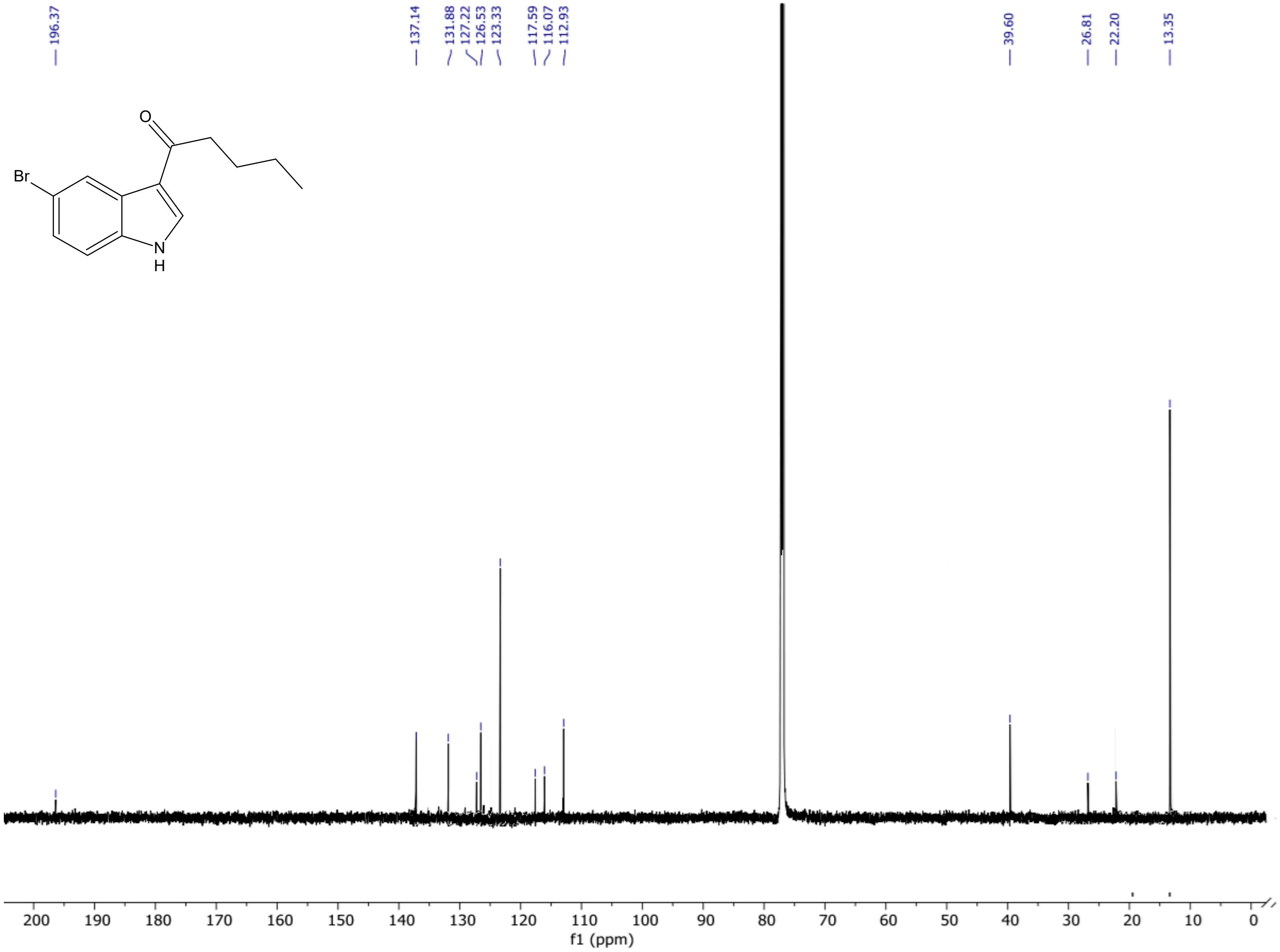

Mass spectra of compound **F**.

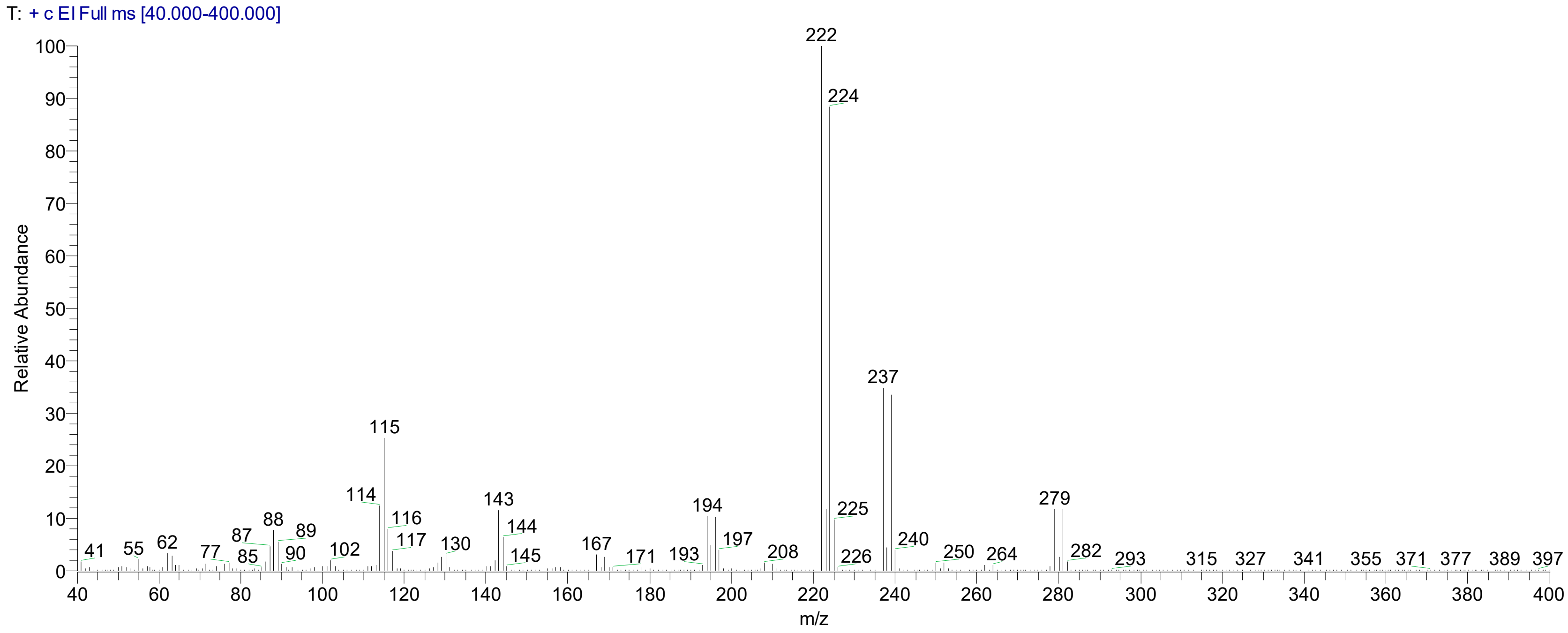

^{1}H NMR spectrum of compound **G**.

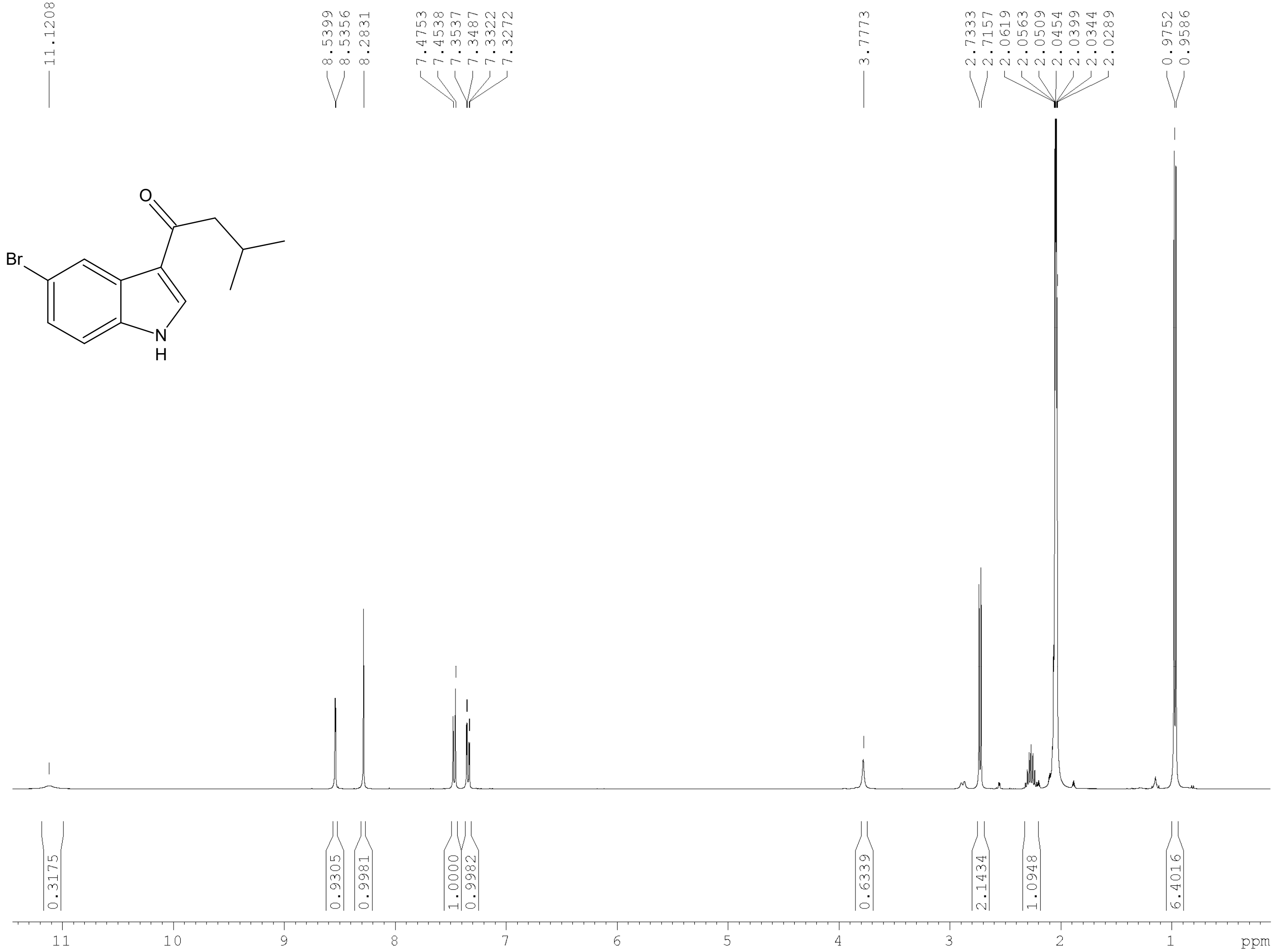

Mass spectra of compound **G.**

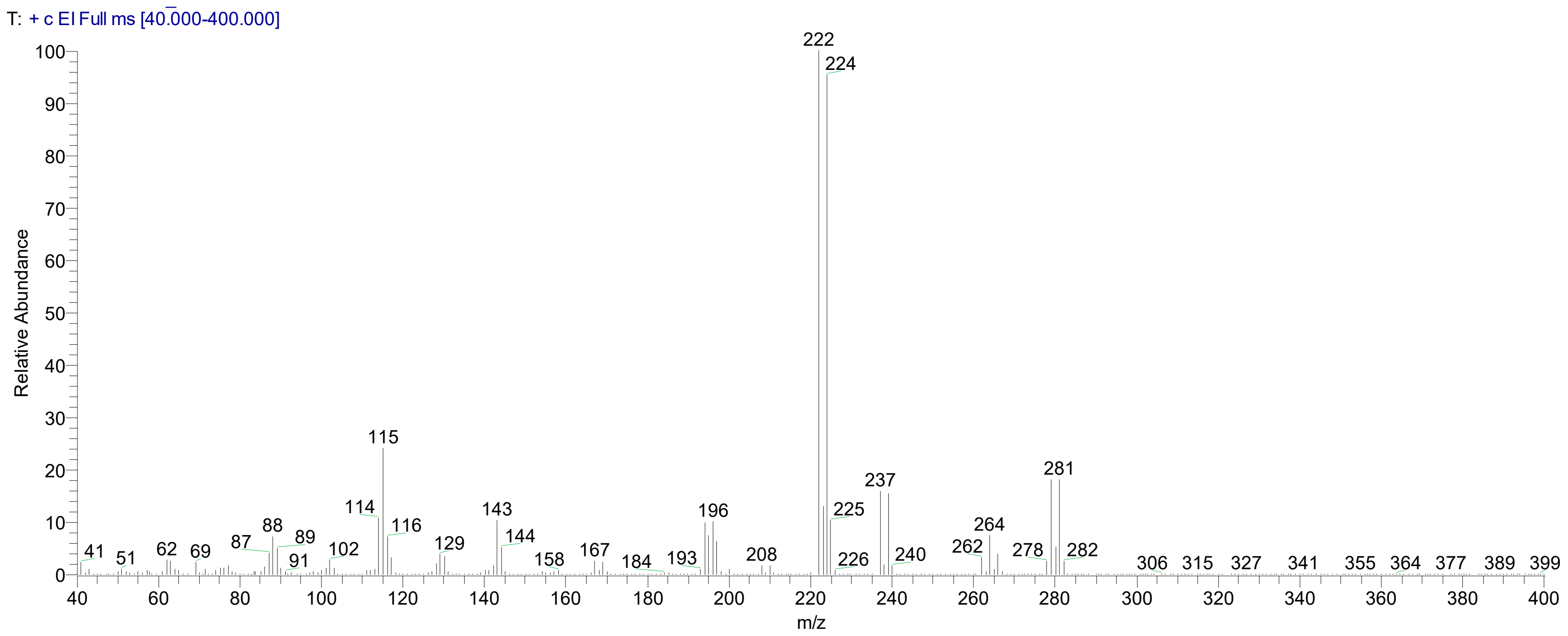

^{1}H NMR spectrum of compound **H**.

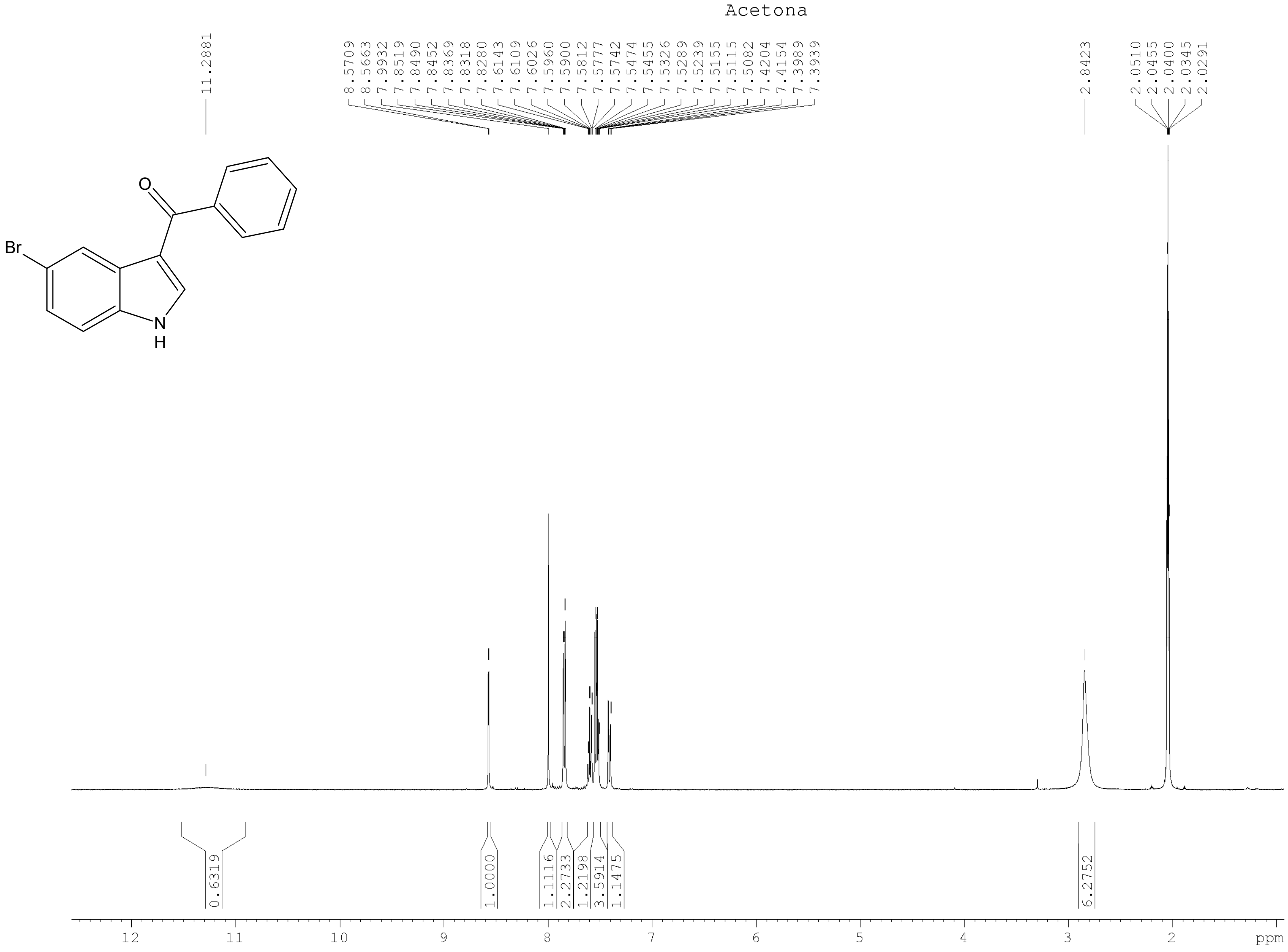

Mass spectra of compound **H**.

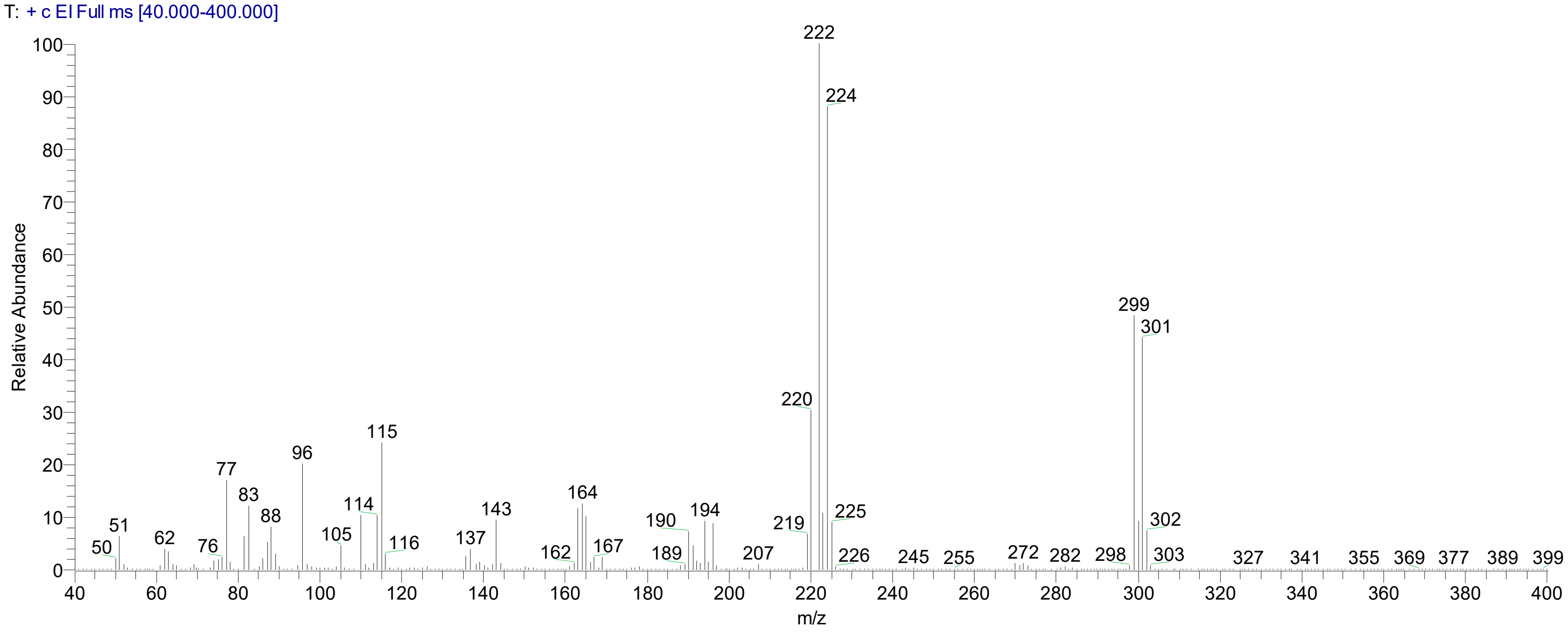

^{1}H NMR spectrum of compound **I**.

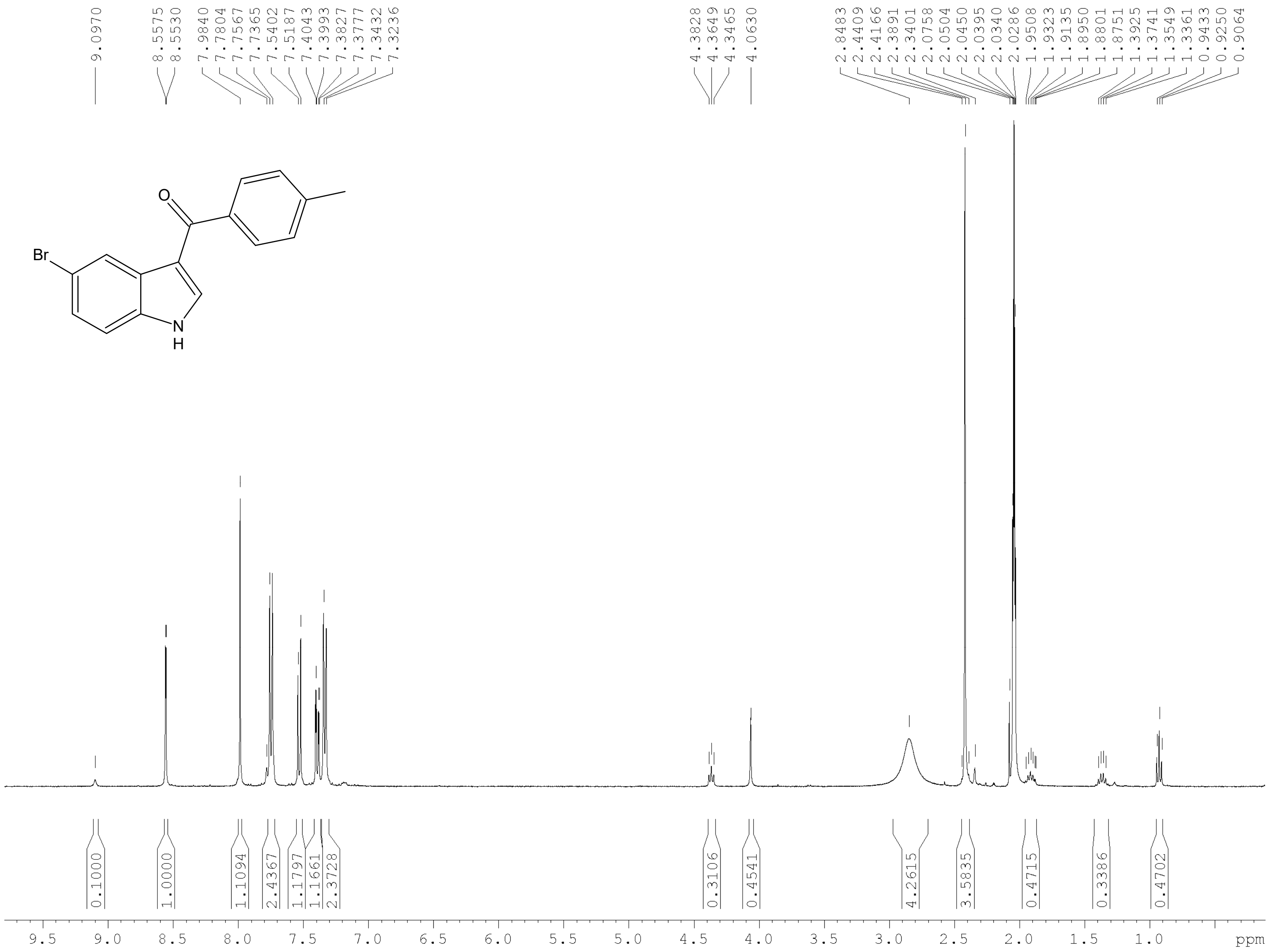

Mass spectra of compound **I**.

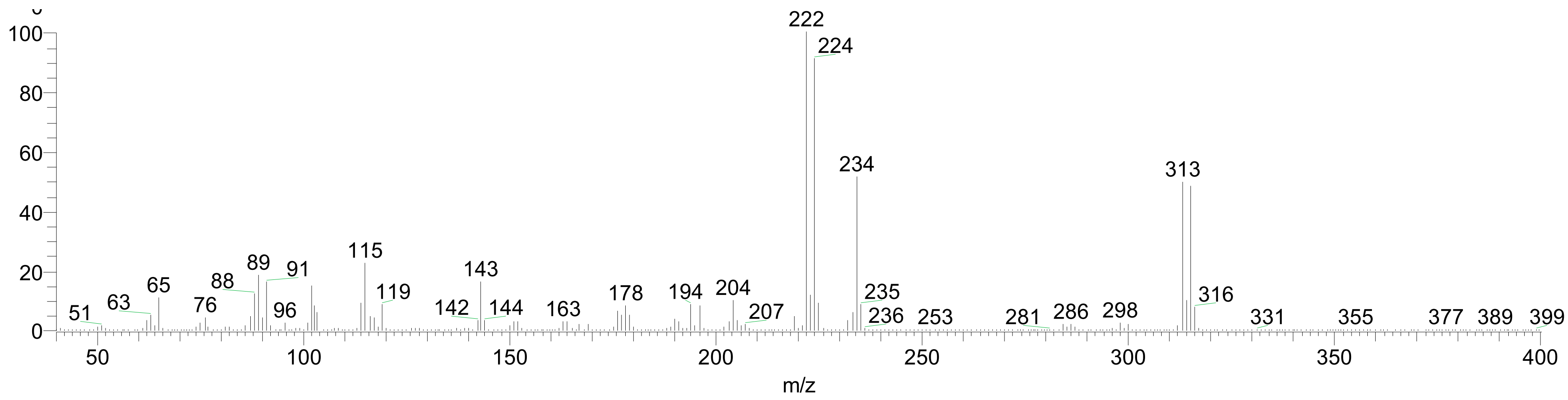

¹H NMR spectrum of compound **J**.

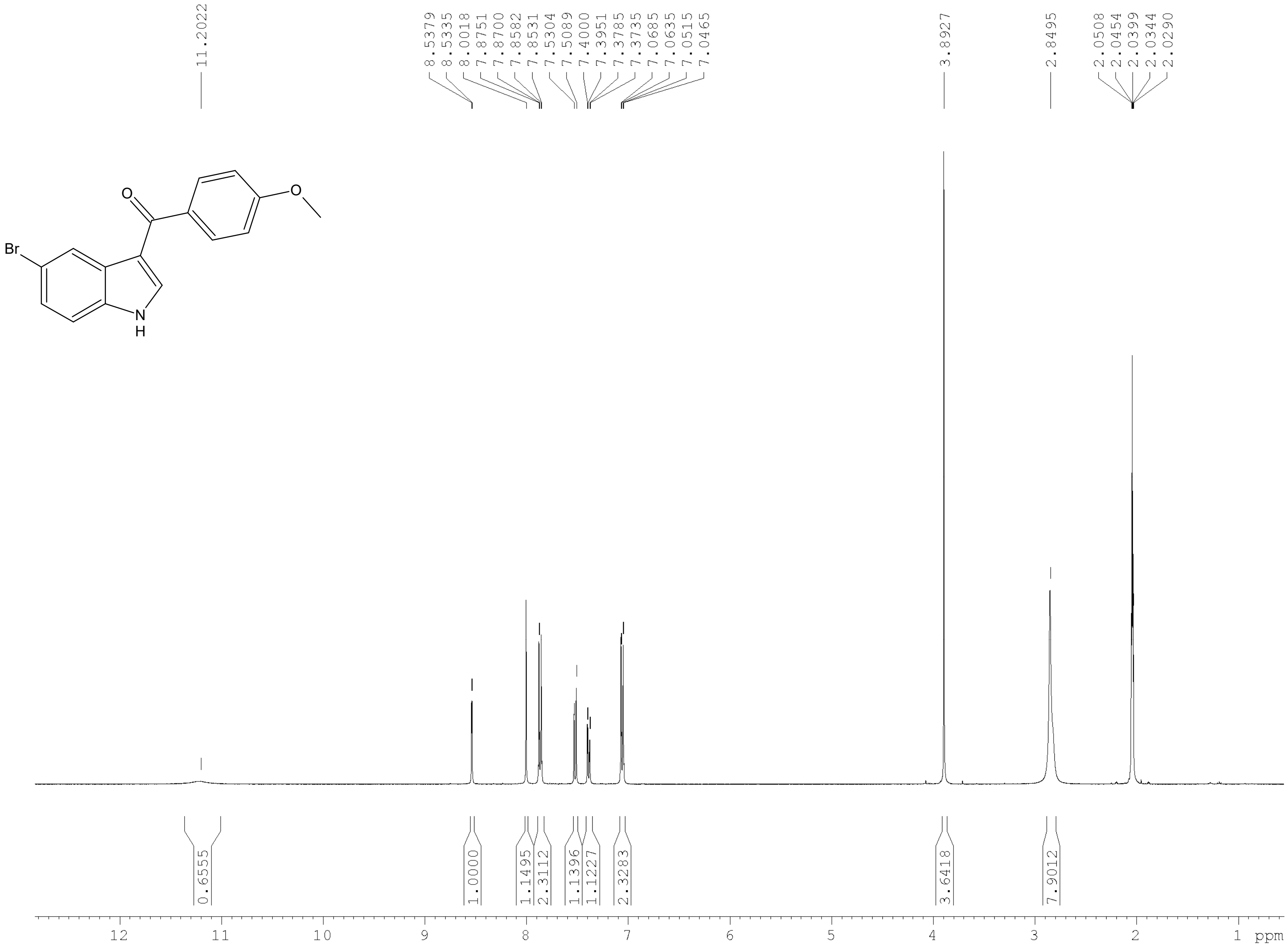

Mass spectra of compound **J**.

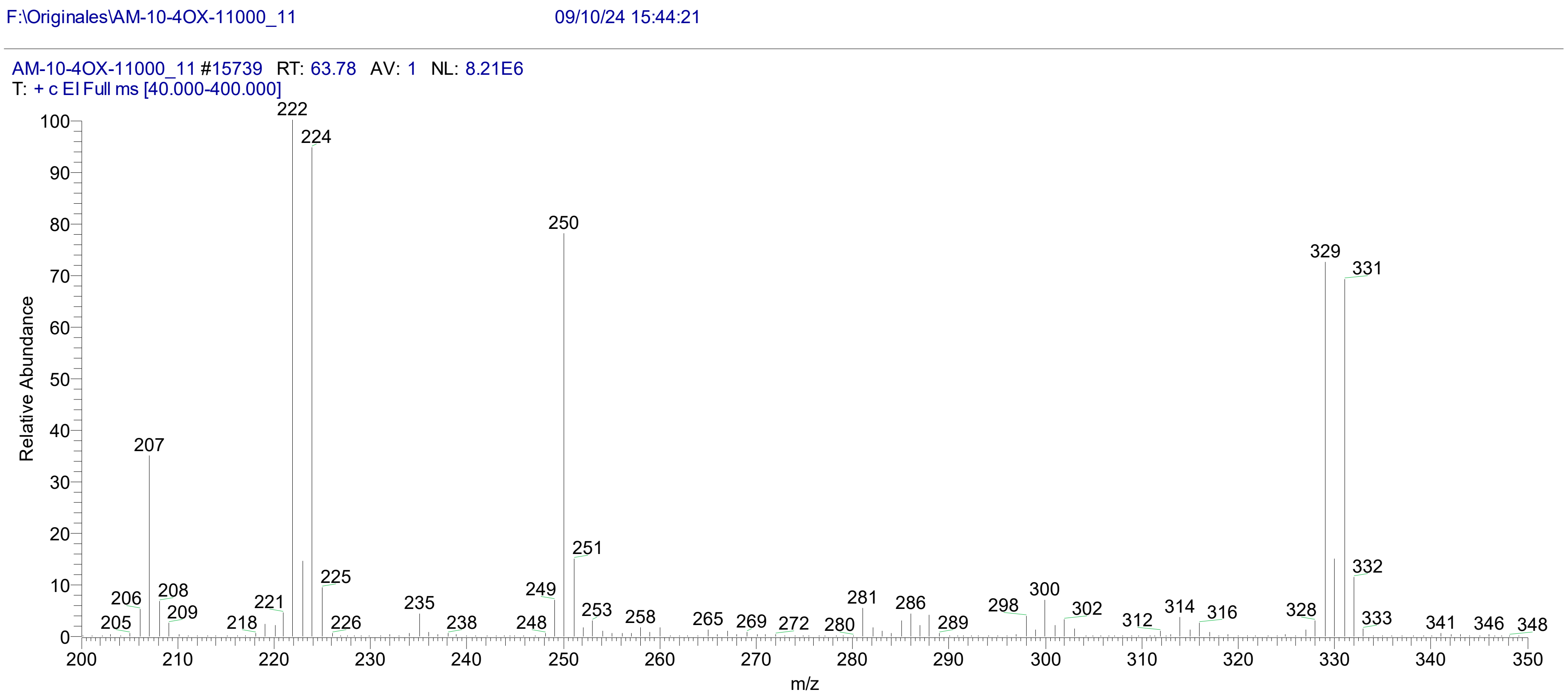

^{1}H NMR spectrum of compound **K**.

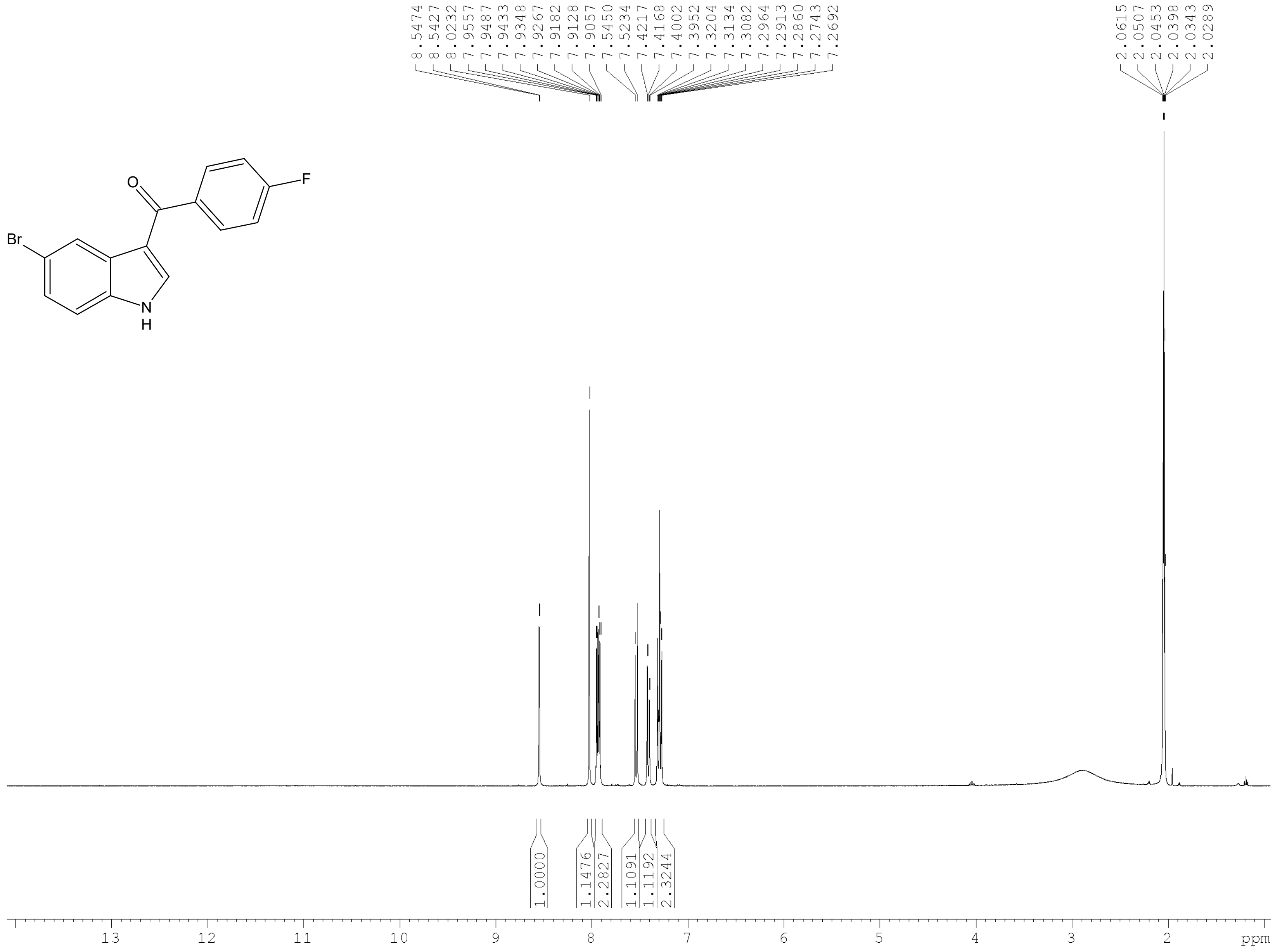

Supplement: Supplementary file 1 [file ijms-26-09148-s001.zip › ijms-3808690-supplementary.pdf]
